# Supplementary figures and images for: Up-regulated chitinase-like protein-1 promotes tumour growth while physiological levels are protective
Source: Life Sci Alliance. 2025 Aug 6;8(10):e202403138. doi: 10.26508/lsa.202403138 (PMC12329362; doi:10.26508/lsa.202403138)

Figure 2 Source data

44 days

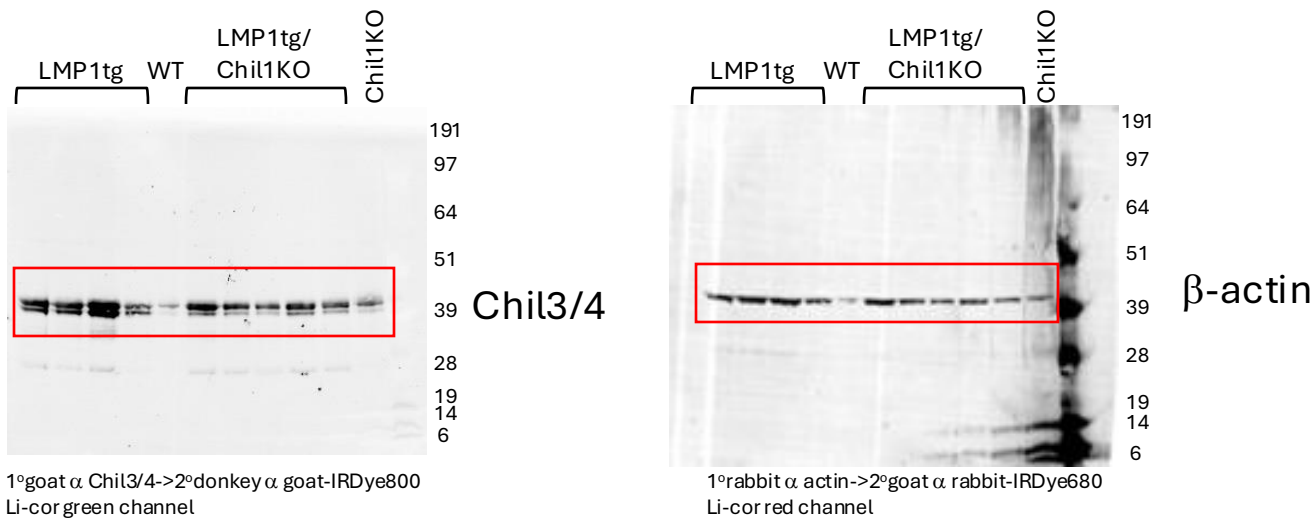

110 days

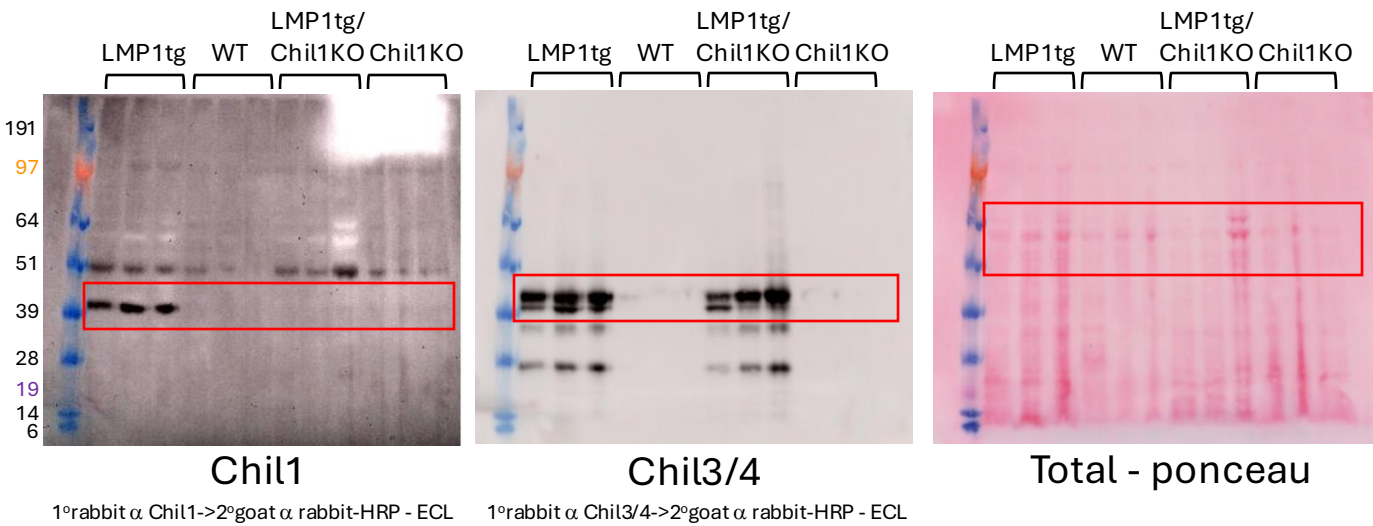

211 days

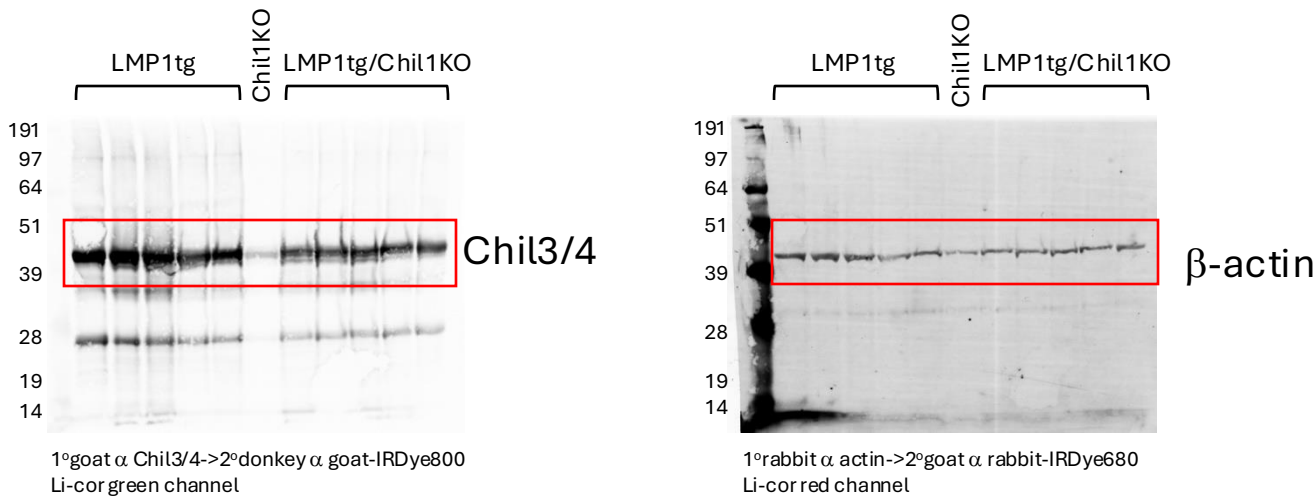

Supplement: Supplementary file 1 [file LSA-2024-03138_SdataF2.pdf]

Figure 5 (D & E ) Source data

D

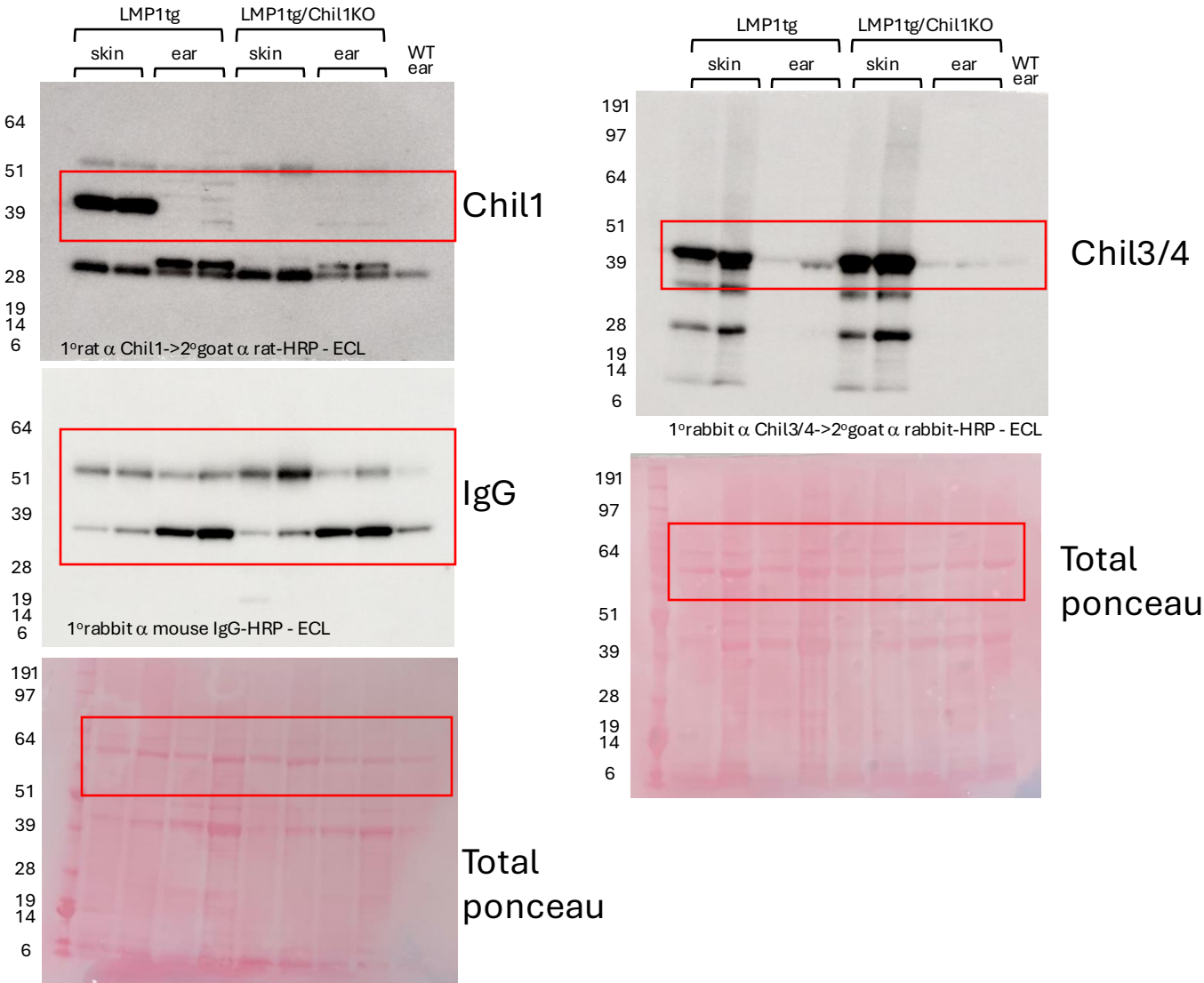

E

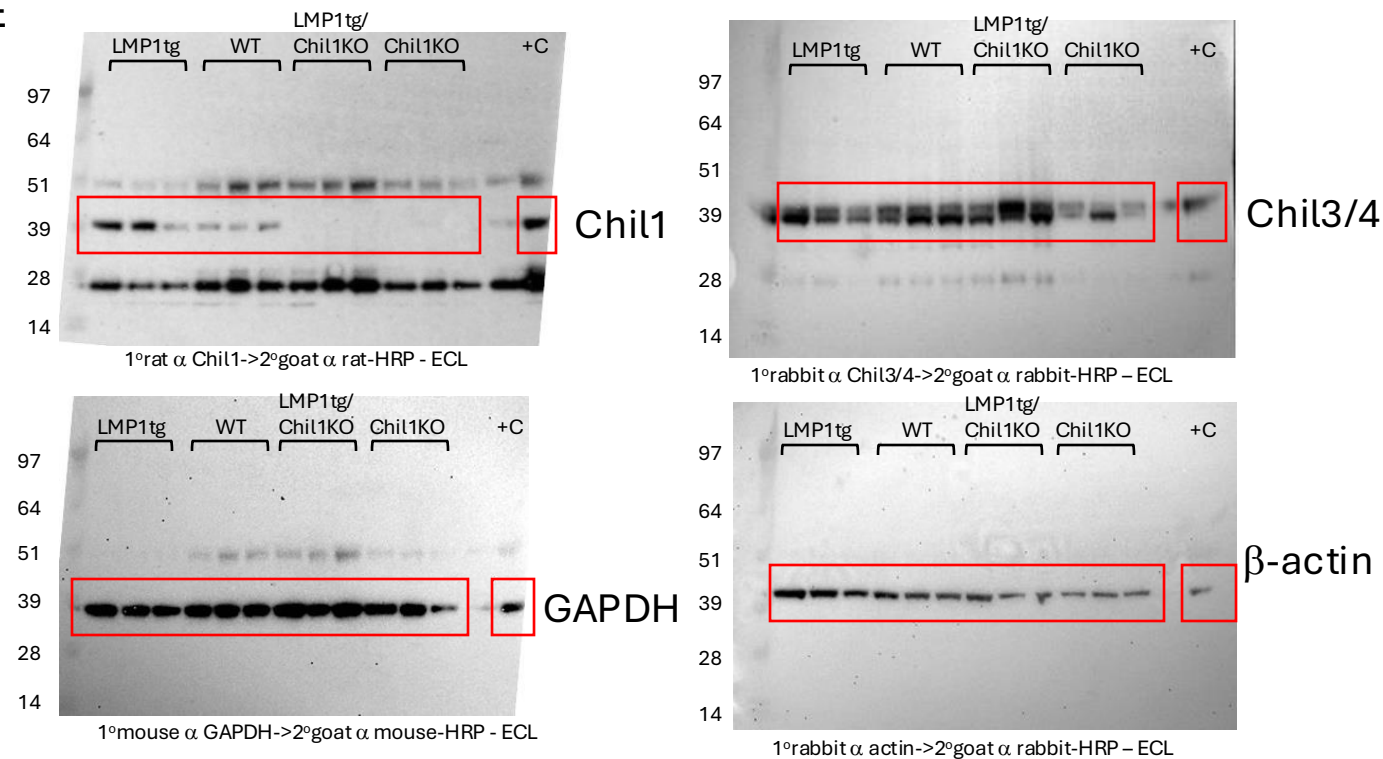

Supplement: Supplementary file 2 [file LSA-2024-03138_SdataF5.pdf]
